# Concentrated Secretome of Adipose Stromal Cells Limits Influenza A Virus-Induced Lung Injury in Mice

**DOI:** 10.3390/cells10040720

**Published:** 2021-03-24

**Authors:** Natalia V. Bogatcheva, Michael E. Coleman

**Affiliations:** 1 Division of Pulmonary, Critical Care and Sleep, Department of Medicine, Indiana University School of Medicine, Indianapolis, IN 46202, USA; 2 Theratome Bio, Inc., Indianapolis, IN 46202, USA; mcoleman@theratomebio.com

**Keywords:** adipose stromal cell, secretome, lung injury, influenza, angiopoietin 2, PDL1

## Abstract

Despite vaccination and antivirals, influenza remains a communicable disease of high burden, with limited therapeutic options available to patients that develop complications. Here, we report the development and preclinical characterization of Adipose Stromal Cell (ASC) concentrated secretome (CS), generated by process adaptable to current Good Manufacturing Practices (cGMP) standards. We demonstrate that ASC-CS limits pulmonary histopathological changes, infiltration of inflammatory cells, protein leak, water accumulation, and arterial oxygen saturation (spO2) reduction in murine model of lung infection with influenza A virus (IAV) when first administered six days post-infection. The ability to limit lung injury is sustained in ASC-CS preparations stored at −80 °C for three years. Priming of the ASC with inflammatory factors TNFα and IFNγ enhances ASC-CS ability to suppress lung injury. IAV infection is associated with dramatic increases in programmed cell death ligand (PDL1) and angiopoietin 2 (Angpt2) levels. ASC-CS application significantly reduces both PDL1 and Angpt2 levels. Neutralization of PDL1 with anti-mouse PDL1 antibody starting Day6 onward effectively ablates lung PDL1, but only non-significantly reduces Angpt2 release. Most importantly, late-phase PDL1 neutralization results in negligible suppression of protein leakage and inflammatory cell infiltration, suggesting that suppression of PDL1 does not play a critical role in ASC-CS therapeutic effects.

## 1. Introduction

Respiratory viruses are major contributors to the socioeconomic burden of infectious diseases. Recent pandemic spread of SARS-CoV-2 emphasized critical importance of the development of treatment options for the mitigation of such burden. Unlike SARS-CoV-2, seasonal influenza viruses were long targeted in the contexts of prevention and treatment, and yet in pre-COVID19 era maintained the highest burden of the communicable diseases, significantly exceeding the burden of tuberculosis and HIV/AIDS [1]. This high burden is attributed to the high incidence and relatively high mortality associated with influenza infection, specifically in the elderly [1]. Even though currently circulating viral strains remain susceptible to the currently used antivirals, severe influenza accounted for 9.4 million cases globally in 2017 [2]. Depending on the circulating strain severity and local demographic situation, up to 23% of hospitalized patients may require admission to intensive care unit (ICU), mostly due to respiratory failure [3]. Viral acute respiratory distress syndrome (ARDS), similar to ARDS of other etiology, is associated with lengthy stay in the ICU, high mortality, and long-term life quality impairment in survivors. Currently available antivirals, including recently approved baloxavir, were only proven to attenuate malaise if applied within a short therapeutic window [4], which has closed in most patients presenting with symptoms of lower respiratory infection. Importantly, meta-analysis showed that even early administration of oseltamivir did not decrease the likelihood of H1N1pdm09-related pneumonia [5]. Other treatment approaches, including immunomodulatory corticosteroids, macrolides and statins, did not lower the risk of severe disease development following H1N1pdm09 infection [6]. There remains an unmet medical need for the development of therapy applicable to patients with an ongoing viral pneumonia/ARDS.

Over the past two decades interest in the therapeutic application of mesenchymal stromal cells (MSC) to a wide variety of pathological conditions, including acute lung injury/ARDS, has increased dramatically [7]. Pathophysiological mechanisms leading to respiratory failure in ARDS include accumulation of pulmonary fluid due to inflammation and dysfunction of endothelial and epithelial barriers, as well as the disruption of fluid-clearing mechanisms in injured resident cells [8]. The complexity of injurious mechanisms in ARDS posits the need for therapy with diverse beneficial effects. Multifaceted anti-inflammatory, immunomodulatory, and reparative properties of MSC supported basis for multiple preclinical and clinical studies [9]. In pre-COVID19 era, bone marrow-derived MSC (BM-MSC) [10], adipose stromal cells (ASC) [11] and menstrual blood-derived [12] stromal cells reached clinical trials and showed good safety profile in ARDS of different etiology. Not surprisingly, COVID19 instigated multiple novel trials of MSC and MSC-derived products in SARS-CoV-2-induced ARDS [7,13,14,15].

Surprisingly, studies of MSC in the narrower context of influenza-induced ARDS generated conflicting results at the earlier preclinical stage. While several groups reported beneficial effects of MSC therapeutic administration in the animal models of highly pathogenic avian influenza H5N1 [16,17] and H9N2 [18], and in an open-label clinical trial in H7N9 ARDS [12], experiments with rodent models of seasonal influenza showed little beneficial effect [19,20]. However, it is wide-spread seasonal influenza which remained the major driver of influenza-associated mortality during the last 100 years [2]; therefore, desirable therapeutic approaches should not be limited to highly pathogenic avian strains.

Continuing translation of cellular therapy to clinical trials, including ARDS trials, revealed that clinical results with cells have been encouraging although not uniformly positive [7]. Whereas some studies showed no significant improvement in clinical outcomes such as 28-day mortality [10], others found improvement in oxygenation and reduction in lung injury parameters [21], with many other clinical trials still ongoing [7]. Among reasons for the “short-of-expectation” clinical outcomes, variation in MSC viability following thawing procedure was discussed [10]. Logistics for widespread clinical application dictate the delivery of cryopreserved and thawed cells, which have been shown to have reduced viability, induced apoptosis, lowered adherence capability, and impaired metabolic activity and immunomodulatory potential [22]. In addition, use of cryopreserved cell therapeutics is limited to the hospitals with access to specific infrastructure, such as liquid nitrogen storage and personnel trained in cell handling. Given that numerous preclinical studies have shown that therapeutic effect of stromal cells is mediated by the secreted paracrine factors [9], the development of a concentrated cell-free product based on MSC secretome could address the logistical limitations of cellular therapy while preserving the multi-factorial nature of the therapeutic mechanism of action.

Here, we report the development of an ASC-based cell-free product efficiently suppressing lung injury in the model of murine lung infection with the seasonal H1N1 influenza. The product is produced by a scalable process that can be carried out in compliance with cGMP to facilitate its translation to clinical practice. The manufacturing process yields good reproducibility of ARDS-limiting potency of preparations. The product has sustained in vivo potency when stored for 3 years at −80 °C. Importantly, the product has shown efficiency when first administered during ongoing lung injury, which increases its relevance to the clinical scenario of ARDS. The study of beneficial mechanisms involved in lung protection revealed the suppression of release of endothelial barrier disruptor Angpt2 along with the suppression of hyperpermeability and inflammation in IAV-injured lung.

## 2. Materials and Methods

### 2.1. Cell Culture

Human pulmonary artery endothelial cells (HPAEC) were purchased from Lonza (Basel, Switzerland) and cultured in EGM2 media (Lonza). When cells in multi-well plate reached 90% confluence, media was changed to basal (EBM2) and supplemented with 20 ng/mL recombinant human TNFα (R&D systems, Minneapolis, MN, USA), 2 u/mL human thrombin (Sigma, St Louis, MS, USA), or 30–100 ng/mL recombinant human VEGF (Sigma). After 24 h incubation, media was collected, and cells were lysed with PBS containing 1% Triton and Pierce antiprotease and antiphosphatase cocktail (Thermofisher Scientific, Waltham, MA, USA). Human Angiopoietin 2 levels in media and cell lysates were determined with human Angpt2 Duo-Set ELISA (R&D systems). Protein levels in cell lysates were determined with bicinchoninic acid (BCA) assay (Thermofisher Scientific).

### 2.2. Preparation of Secretome

ASC secretome was prepared from human ASC derived from lipoaspirate sample of a healthy adult female donor undergoing elective lipoplasty with informed consent. Health status was assessed by preoperative exam and negative test results for infectious diseases. ASC were expanded at Cytovance Biologics (Oklahoma City, OK, USA) in compliance with cGMP to produce a Master Cell Bank, which was tested for adventitious agents, sterility, expression of ASC markers CD73, CD90, and CD105 and absence of markers CD34 and CD45, and tri-lineage differentiation. For production of secretome, working cell bank of human ASC was thawed and plated in cGMP-compliant proprietary growth media at 5 × 10^3^ cells/cm^2^ and expanded in adherent culture. For immune priming, human TNFα and IFNγ (Peprotech, Cranbury, NJ, USA) were added (5 nM each) to growth media for 24 h before conditioning. To produce conditioned media, 70% confluent ASC cultures were washed 3X with sterile PBS, after which media without growth factors, blood products and phenol red was added. ASC were cultured for 60–72 h, and then media were harvested, filtered through 0.45 µM filter, concentrated approximately 100-fold using tangential flow filtration (PES membrane, <5 kDa pore size), filtered through 0.22 µm PES membrane filter, aliquoted, and stored at −80 °C in sterile polypropylene tubes.

### 2.3. Animals

C57Bl/6 strain was chosen for H1N1 infection, as model of lung injury by seasonal influenza is well-described in these mice [19,20]. Adult 12–16-wk-old C57BL/6 mice were ordered from the Indiana University (IU) In vivo therapeutic core. Animals were cared for in accordance with NIH guidelines by the IU Laboratory Animal Resource Center, Indianapolis, and all experiments were conducted under protocols approved by the IU School of Medicine Institutional Animal Care and Use Committee (protocol 1223, approved 2 February 2017, and protocol 19146, approved 27 January 2020). Group size sufficient to ensure adequate statistical power was determined based on our prior experience with the models of IAV-induced acute lung injury [23].

### 2.4. Viral Infection, Administration of Therapeutic Material, and Assessment of Viral Antigen

**Figure 1:**
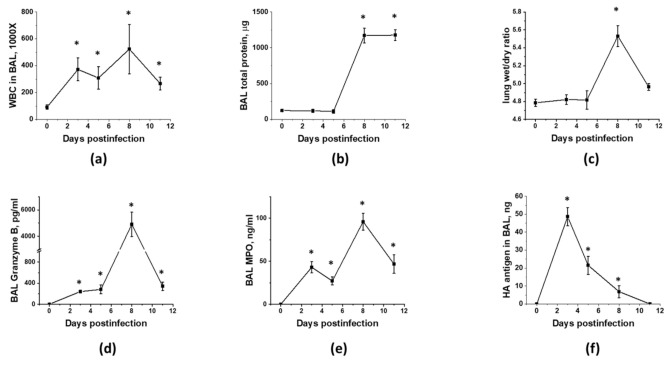
Murine lung infection with IAV is characterized by two distinct phases, a viral clearance phase and a lung injury phase, with white blood cell infiltration during former and latter phases. C57Bl/6 mice received intranasal administration of IAV H1N1 PR8 (800 pfu/mouse) on Day0. Lungs and BAL were collected on the days indicated to analyze for BAL total protein levels (**a**) and white blood cell count (**b**), lung wet/dry ratio (**c**), acellular BAL granzyme B (**d**) and MPO (**e**) levels, and PR8 hemagglutinin level (**f**). *n* = 4–8 per group, presented are mean +/− SEM. * for *p* < 0.05 by *t*-test with Welch correction (unequal variances) between IAV groups and the control group, receiving saline in lung (plotted as D0).

**Figure 2:**
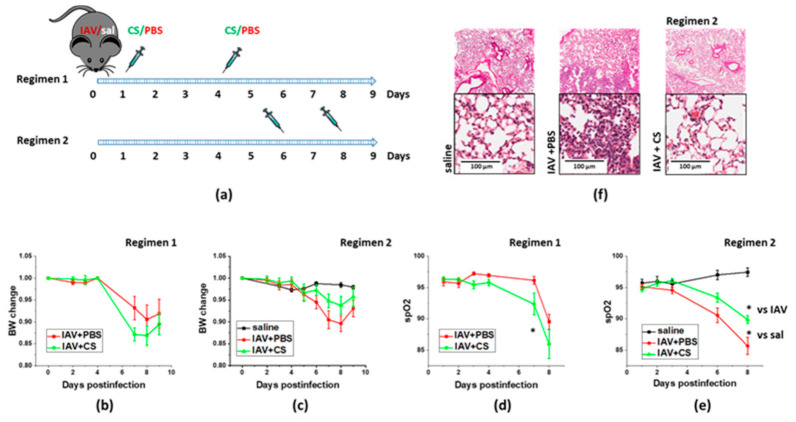
Late ASC-CM administration concomitant with onset of lung injury limits weight loss and lung histopathological changes in response to influenza infection. Schematic of two regimens of therapeutic administration of ASC-CS (**a**). The 12–16-week-old C57Bl/6 mice received intranasal administration of IAV H1N1 PR8 (800 pfu/mouse) on Day0. Control mice received equivalent volume of saline (sal). On the days indicated, mice were sub-cutaneously injected with 2.25 mg/kg of ASC-CS (CS) or an equivalent volume of PBS. Lungs were collected for analyses on Day9. Body weight change in mice subjected to regimen 1 (**b**) or 2 (**c**). Blood oxygenation changes in mice subjected to regimen 1 (**d**) or 2 (**e**). Histopathological changes in H&E-stained lungs from mice subjected to regimen 2 (**f**). Presented are mean +/− SEM. * for *p* < 0.05 by *t*-test with Welch correction (unequal variances).

**Figure 3:**
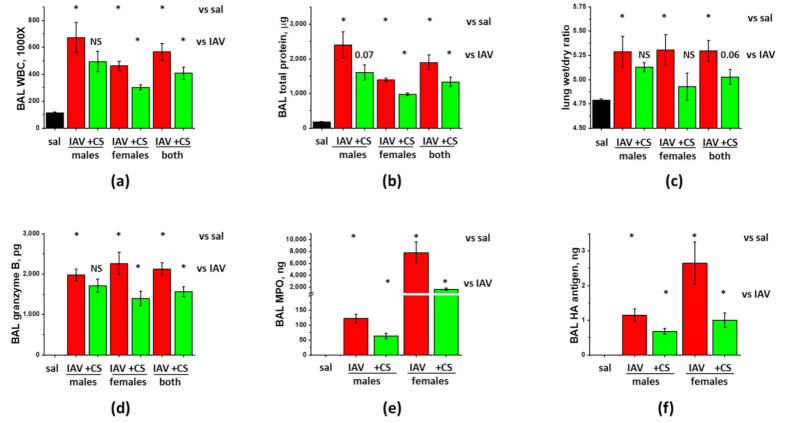
ASC-CS administration limits BAL protein and inflammatory cell extravasation, and increases in granzyme B and MPO without compromise of viral clearance in IAV-infected mice. C57Bl/6 mice received intranasal administration of IAV H1N1 PR8 (800 pfu/mouse) on Day0. Mice receiving vehicle control for IAV are annotated as saline (sal, black columns). On Day6 and 8, IAV mice were sub-cutaneously injected with 2.25 mg/kg of ASC-CS (CS, green columns) or an equivalent volume of PBS (red columns designated as IAV). Lungs and BAL were collected on Day9 to analyze for BAL white blood cell count (**a**) and total protein levels (**b**), lung wet/dry ratio (**c**), acellular BAL granzyme B (**d**) and MPO (**e**) levels, and PR8 hemagglutinin (HA) level (**f**). *n* = 4–6 per group, presented are mean +/− SEM. * for *p* < 0.05 by *t*-test with Welch correction (unequal variances) between (1) IAV groups and the control group (vs. sal), and (2) CM groups and the corresponding gender IAV group (vs. IAV).

**Figure 4:**
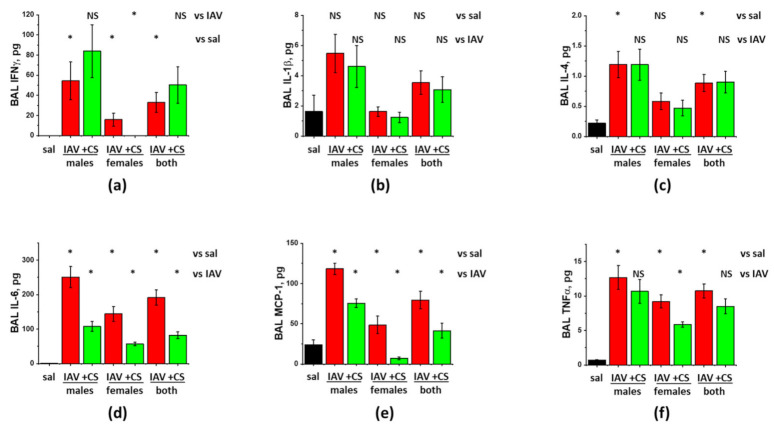
ASC-CS administration limits inflammatory cytokine increases in BAL of IAV-infected mice. C57Bl/6 mice received intranasal administration of IAV H1N1 PR8 (800 pfu/mouse) on Day0. Mice receiving vehicle control for IAV are annotated as saline (sal, black columns). On Day6 and 8, IAV mice were sub-cutaneously injected with 2.25 mg/kg of ASC-CS (CS, green columns) or an equivalent volume of PBS (red columns designated as IAV). BAL fluids were collected on Day9 to analyze for IFNy (**a**), IL-1b (**b**), IL-4 (**c**), IL-6 (**d**), MCP-1 (**e**), and TNFα levels (**f**). *n* = 4–6 per group, presented are mean +/− SEM. * for *p* < 0.05 by *t*-test with Welch correction (unequal variances) between (1) IAV groups and the control group (vs. sal), and (2) CS groups and the corresponding gender IAV group (vs. IAV). NS - differences between the groups are not significant.

**Figure 5:**
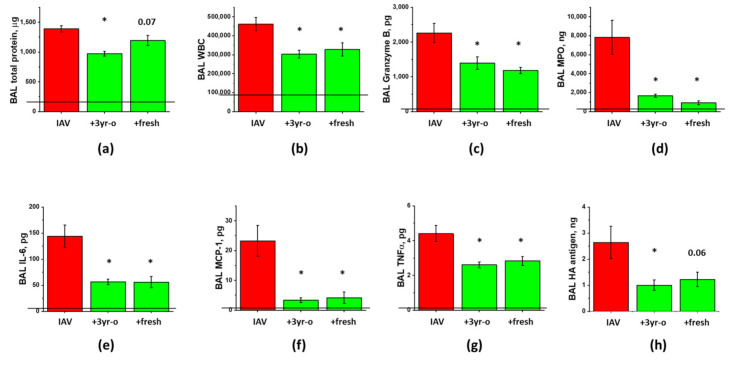
Long-term storage at −80 °C does not compromise ASC-CS ability to limit lung injury in IAV-infected mice. Female C57Bl/6 mice received intranasal administration of IAV H1N1 PR8 (800 pfu/mouse) on Day0. On Day6 and 8, IAV mice were sub-cutaneously injected with 2.25 mg/kg of fresh or 3 year-old (3y-o) ASC-CS (green columns) or an equivalent volume of PBS (red columns). BAL fluids were collected on Day9 to analyze for total protein levels (**a**), white blood cell counts (**b**), granzyme B (**c**) and myeloperoxidase levels (**d**), IL-6 (**e**), MCP-1 (**f**) and TNFa levels (**g**), and PR8 hemaggutinin (HA) levels (**h**). *n* = 4–5 per group, presented are mean +/− SEM. Lines indicate levels of analytes detected in control (saline to lung) mice. * for *p* < 0.05 by *t*-test with Welch correction (unequal variances) between CS groups and IAV group.

**Figure 6:**
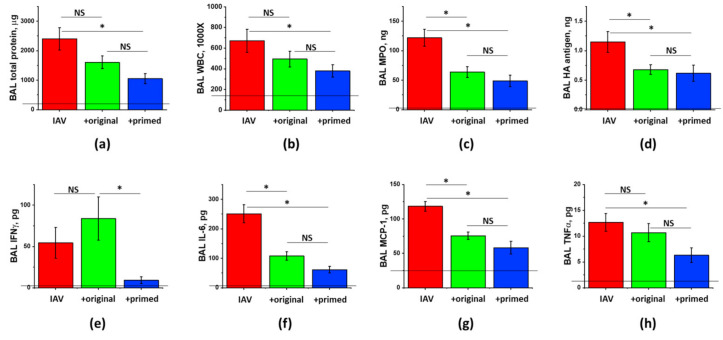
Immune priming of ASC improves ASC-CM ability to limit hyperpermeability and inflammation in IAV-infected mice. Male C57Bl/6 mice received intranasal administration of IAV H1N1 PR8 (800 pfu/mouse) on Day 0. On Day6 and 8, IAV mice were sub-cutaneously injected with 2.25 mg/kg of the original (green columns) or primed (blue columns) ASC-CS or an equivalent volume of PBS (red columns). BAL fluids were collected on Day9 to analyze for total protein levels (**a**), white blood cell counts (**b**), myeloperoxidase (**c**) and PR8 hemagglutinase levels (**d**), and IFNγ (**e**), IL-6 (**f**), MCP-1 (**g**) and TNFα (**h**) levels. *n* = 4–6 per group, presented are mean +/− SEM. Lines indicate levels of analytes detected in control (saline to lung) mice. * for *p* < 0.05 by *t*-test with Welch correction (unequal variances) between groups indicated. NS - differences between the groups are not significant.

IAV H1N1 PR8 (American Type Culture Collection (ATCC), Manassas, VA, USA) was aliquoted and stored at −80 °C. To improve reliability across experiments, all working dilutions of virus were prepared from the freshly thawed aliquots of the same ATCC batch. Mice were anesthetized deeply with isoflurane and inoculated intranasally with 800 pfu/mouse in 60 uL of sterile saline according to the previously established protocol [23]. Control mice received equivalent saline volume. For time course experiment, mice were terminated at times indicated in Figure 1. ASC-CS preparations (2.25 mg/kg) were administered subcutaneously (SQ) at times indicated in Figures 2–6 legends; equivalent volume of sterile PBS was delivered to “virus only” animals. Dose was chosen based on the prior dose–response studies in the murine ischemic stroke models (dose range 0.2–5.0 mg/kg body weight (Dr. Coleman, unpublished observations)). Anti-mouse PDL1 antibody (GoInVivo clone 10F.9G2, Biolegend, San Diego, CA, USA) were administered SQ (8 mg/kg) at Day 6 and Day 8 post-infection. Mice were terminated at Day 9, at the peak of lung injury [23]. Postmortem viral antigen levels in bronchoalveolar lavage (BAL) were measured using PR8 hemagglutinin (HA) ELISA (Sino Biological, Wayne, PA, USA).

### 2.5. Blood Oxygenation Monitoring

Pulse oximetry was performed in shaved and acclimated conscious mice using the MouseOx cervical collar sensor (Starr Life Sciences, Oakmont, PA, USA), as described previously [23]. Arterial oxygen saturation (spO_2_) was monitored for 5–10 min at each day of assessment.

### 2.6. Analysis of Lung Injury Indices

Mice were placed under general anesthesia with isoflurane, a bilateral thoracotomy was performed, and then animals were exsanguinated via right ventricular puncture. The left lung was clamped for wet/dry ratio determination. The right lung was flushed with excess of ice-cold PBS. BAL fluid was collected from the right lung by delivering 3 flushes of 0.8 mL of ice-cold PBS via cannulated trachea. BAL white blood cell (WBC) count was assessed after treating pelleted cells with Red Blood Cell lysis buffer. BAL protein level was measured in acellular BAL using Bradford assay (BioRad, Hercules, CA, USA). For histology, the lungs were fixed in 4% paraformaldehyde and paraffin embedded. Five micron sections were stained with hematoxylin-eosin. BAL cytokines were measured using a mouse inflammatory 10-plex Discovery assay by Eve Technologies (Calgary, AB, Canada). BAL myeloperoxidase (MPO) and Granzyme B levels were measured using mouse-specific duo-set ELISA from R&D Systems.

### 2.7. Analysis of Angiopoietin 2 (Angpt2) and PDL1 Pathway

BAL fluid was analyzed with mouse Angpt2 quantikine ELISA (R&D Systems) and mouse PDL1 Duo-set ELISA (R&D Systems). Lung was extracted with 1%Triton X-100 in PBS containing Pierce anti-protease and anti-phosphatase cocktail (ThermoFisher scientific) and analyzed with mouse PDL1 ELISA, mouse p-Tie2 Duo-set ELISA (R&D systems) and mouse Tie2 quantikine ELISA (R&D systems).

### 2.8. Statistical Analyses

All analyses other than recording of body weight and lung wet and dry weight were done at least in duplicate. Data points lying outside of 1.5 of interquartile range were considered outliers. Comparisons between groups were done with unpaired *t* test with Welch correction (for groups with different variance). Statistical analysis and graphing were performed with Origin 16 (OriginLab, Northhampton, MA, USA).

## 3. Results

### 3.1. Optimization of the Timeline for Therapy Administration

To facilitate testing of both “early” and “late” therapeutic intervention protocols, tests were conducted in the non-lethal model of IAV PR8 infection. Previous tests of BM-MSC in IAV PR8 model included pre-infection, co-infection, and early and late post-infection protocols, with some suppression of IAV-induced thrombocytosis reported but no effect on acute lung injury [19,20]. For our experiments, subcutaneous (SQ) administration was chosen due to the following reasons: (a) in murine models, SQ delivery has lesser chance of missing target delivery route than intravenous delivery; (b) SQ delivery was previously shown to successfully reduce targeted mediators in lung [23]; (c) plasma concentrations of SQ-delivered biologic show more uniform non-spiking pharmacokinetic patterns in contrast to intravenously delivered biologic [24].

As MSC are known for their immunomodulatory properties, and immune cell infiltration to lung is driving both detrimental lung injury and beneficial pathogen clearance, we next investigated the temporal relationship between viral replication, immune cell infiltration, and the development of ARDS. As shown in Figure 1, immunocompetent mice display two distinct phases of immune cell infiltration (Figure 1a) in response to inoculation of the non-lethal dose of IAV to lung. First phase coincides with viral replication (Figure 1f) and is accompanied by the detectable release of the Granzyme B (Figure 1d), a component of NK and T cell cytotoxic granules, and MPO (Figure 1e), a Reactive Oxygen Species (ROS)-generating enzyme. Despite increased Granzyme B and MPO levels, phase 1 is not accompanied by increases in lung fluid accumulation or protein extravasation to BAL (Figure 1b,c). Second phase of immune cell infiltration is characterized by higher levels of Granzyme B and MPO in BAL, and distinct changes in barrier permeability indices and spO2 (Figure 2d,e), evident of lung injury. Importantly, these changes are seen in the presence of viral antigen levels markedly lowered in comparison to levels seen in phase 1. As expected from the non-lethal dose, both viral infection and lung injury are self-resolving in our model.

We next investigated whether timing of therapeutic material administration would result in different outcomes in the context of IAV-induced lung injury. We have compared a protocol utilizing early start of CS administration (regimen 1, Figure 2a) with the protocol limiting administration to the second phase of malaise (regimen 2, Figure 2a). IAV-infected mice subjected to regimen1 manifested more dramatic weight loss, and significantly greater decrease in spO2 levels than infected mice subjected to vehicle administration (Figure 2b,d). In contrast, infected mice subjected to the regimen2 manifested limited weight loss and significantly attenuated decrease in spO2 levels when compared to mice subjected to the vehicle administration (Figure 2c,e). Histological analysis of lung from IAV-infected mice examined at D9 post-infection revealed areas of lung tissue consolidation consistent with the focal interstitial pneumonia (Figure 2f). Lungs from IAV-infected mice subjected to late ASC-CS administration revealed areas with minor patches of lung tissue consolidation along with areas with no apparent consolidation. Based on these findings, regimen 2 was chosen to further test ASC-CS preparations in the model of IAV-induced lung injury.

### 3.2. Sex-Dependent Responses to ASC-CS in the Model of IAV-Induced Lung Injury

Since both biological and sociocultural factors differentially affect exposure and severity of influenza as well as responses to available prophylactic and therapeutic measures [25], we investigated the ability of ASC-CS to mitigate IAV-induced lung injury in both female and male C57Bl/6 mice. The tests were conducted in accordance with the above-described protocol, when subcutaneous CS administrations were carried out at D6 and D8 post-infection. As seen in Figure 3a–c, both males and females respond to IAV infection with comparable increases in BAL WBC infiltration, protein extravasation and lung fluid accumulation. Increases in inflammatory cytokines IL-6, MCP-1, and TNFα were also of comparable value between males and females (Figure 4). Surprisingly, while BAL Granzyme B levels were almost identical in IAV-infected males and females (Figure 3d), BAL MPO levels were dramatically higher in females (Figure 3e). Detected levels of viral antigen were also higher in females (Figure 3f).

Analysis of the effect of ASC-CS administration showed that both WBC infiltration and protein extravasation were significantly suppressed in IAV-infected female-only, but not male-only groups (Figure 3a,b). When the results from the mixed-gender group were analyzed, reduction in both WBC infiltration and protein extravasation was found significant. Analysis of the inflammatory markers showed significant suppression of IL-6 and MCP-1 levels in both female-only and male-only groups (Figure 4c,d). IFNγ and TNFα levels were suppressed in female-only group (Figure 4a,f); suppression in mixed-gender group was not significant. Granzyme B levels were significantly reduced in female-only and mixed gender group (Figure 3), whereas MPO levels were reduced in both genders. Importantly, viral antigen levels were significantly lower in both female-only and male-only group. Altogether, these results show that late-phase intervention with ASC-CS significantly suppresses ARDS indices in mixed-gender mice, with the female mice being more susceptible to the beneficial effects of the ASC-CS. Importantly, the suppression of the inflammatory cell response during late phase of IAV infection by ASC-CS was not accompanied by compromise of the viral clearance.

### 3.3. The Effect of the Long-Term Storage at −80 °C on ARDS-Mitigating Potency of ASC Secretome Preparations

One of the advantages of secretome preparations versus cell therapies is the lack of necessity for storage in liquid nitrogen gas phase or ultralow temperature freezer at <130 °C. To ascertain product stability at −80 °C, we next tested the oldest available preparation generated with the same protocol and stored for three years. To test whether long storage rather than freezing-thawing negatively affected the ability of secretome preparation to suppress IAV-induced lung injury, three-year old preparation was compared to freshly generated preparation which was kept at −80 °C for a short period of time (two weeks). The experiment was carried out in females, to facilitate potential detection of the inferior effect of the three year-old preparation.

Comparison of the effects of freshly generated and stored-for-3-years preparations showed that both preparations were similarly effective in suppressing the inflammatory cell infiltration, the levels of inflammatory markers, and the release of cytotoxic Granzyme B and ROS-generating enzyme MPO (Figure 5). The suppression of the total protein extravasation, and the improvement of antigen clearance were more pronounced in mice treated with the stored-for-3-years preparation. Altogether, these results indicate that manufacture of concentrated ASC secretome according to cGMP-compliant protocol yields preparations of reproducible ARDS-mitigating potency, and that three year-long storage does not significantly affect the ability of CM frozen at −80 °C to elicit lung-protective effects.

### 3.4. The Effect of ASC Inflammatory Priming on the ARDS-Mitigating Potency of ASC Secretome Preparations

Data of literature describe that short-term inflammatory priming of BM-MSC with inflammatory cytokines such as TNFα, IFNγ, and IL1β results in the potentiation of their anti-inflammatory properties [26,27]. To explore whether inflammatory priming of ASC would yield secretome with enhanced ability to mitigate ARDS, we compared therapeutic potency of the ASC-CS preparations generated from the same-donor ASC according to standard protocol or a modified protocol including immune priming with TNFα and IFNγ, followed by the subsequent inflammatory cytokine removal before conditioning. The comparison was carried out in male-only groups, to facilitate potential detection of the superior effect of the enhanced secretome and to determine whether ARDS mitigation can be improved in the gender which is more therapy-resistant.

Analysis of ARDS indices in infected male mice had shown significant attenuation of protein extravasation and WBC infiltration into BAL by inflammatory-primed, but not the original, secretome preparation (Figure 6a,b). BAL TNFα levels were also reduced by immune-primed, but not the standard secretome (Figure 6h). These data suggest that immune priming is able to further enhance the potential of ASC-CS to reduce lung inflammation in viral ARDS. As we had shown earlier, suppression of inflammation by secretome preparations is not associated with the compromise of viral clearance (Figure 6d).

### 3.5. IAV-Induced Lung Injury Is Associated with Angpt2 Release, Which Can Be Suppressed by ASC Secretome

**Figure 7:**
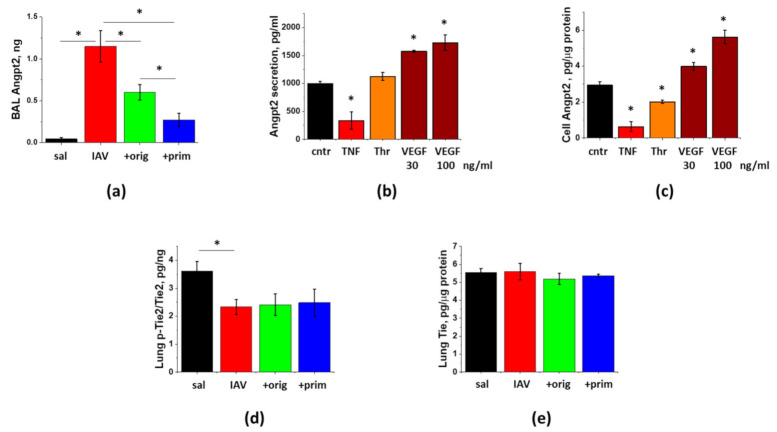
Lung injury is associated with the release of Angpt2, which can be decreased by ASC-CS. Mice from Figure 6 (saline to lung- black; IAV + PBS—red; IAV + original ASC-CS—green; IAV + primed ASC-CS—blue) were terminated at Day9 to analyze for BAL Angpt2 (**a**) and lung p-Tie2 (**d**) and Tie2 (**e**) levels. *n* = 4–6 per group, presented are mean +/− SEM. * for *p* < 0.05 by *t*-test with Welch correction (unequal variances) between groups indicated. HPAEC were stimulated with 20 ng/mL TNFα, 2 unit/mL Thrombin, and 30 and 100 ng/mL VEGF (**b**,**c**). After 24 h, media (**b**) and cell extracts (**c**) were collected and analyzed. Levels of Angpt2 were normalized per protein levels in cell extracts. Mean +/− SEM from 3 independent experiments are presented. * for *p* < 0.05 by *t*-test with Welch correction (unequal variances) when compared with control.

To determine whether Angpt2 release contributes to the pathogenesis of IAV-induced lung injury, we assessed the levels of Angpt2 in BAL of infected mice. Figure 7a shows dramatic induction of Angpt2 levels at Day9 post-infection. We next tested whether direct stimulation of endothelial cell by IAV would result in Angpt2 release. Stimulation of HPAEC with 1 MOI IAV for 4 h or 24 h did not yield increase in Angpt2 release or synthesis (data not shown). Application of other lung injury-relevant factors shown to stimulate endothelial Angpt2 secretion or expression [28,29,30] gave disparate results in HPAEC. TNFα showed marked and significant suppression of Angpt2 levels in HPAEC and in 24 h conditioned media (Figure 7b,c). Thrombin also demonstrated significant suppression of cellular Angpt2 levels. VEGF dose-dependently up-regulated both Angpt2 secretion and expression in HPAEC.

Analysis of *in vivo* effects of ASC-CM on Angpt2 secretion revealed that mice receiving injections of the original or primed secretome preparation manifested markedly lower levels of Angpt2. Effect of the primed secretome preparation was significantly more prominent than the effect of the original preparation.

To determine whether decrease of Angpt2 release leads to the corresponding increase in phosphorylation of its downstream target p-Tie2, we analyzed lung levels of p-Tie2. Angpt2 release during the lung injury phase of IAV infection was associated with the significant decrease in lung p-Tie2 levels (Figure 7d). Surprisingly, lungs of mice receiving ASC secretome administration did not demonstrate increase in p-Tie2 levels concomitant with the suppression of Angpt2 release. The total levels of Tie2 protein in lung remain unchanged in response to the infection or the administration of secretome (Figure 7e).

### 3.6. PDL1 Ablation during Lung Injury Phase Does Not Attenuate Angpt2 Release, Protein Leak, or Inflammation in IAV-Infected Mice

**Figure 8:**
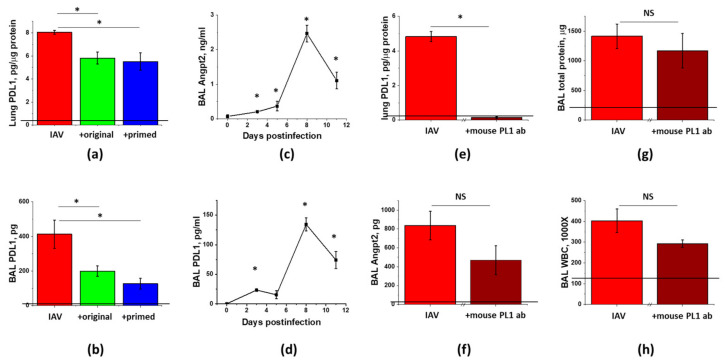
Levels of PDL1 and Angpt2 are affected by IAV infection and ASC-CS therapy in a similar manner; however, late stage ablation of PDL1 does not significantly attenuate Angpt2 levels or lung injury. Mice from Figure 6 (IAV + PBS—red; IAV + original ASC-CS—green; IAV + primed ASC-CS—blue) were terminated at Day9 to analyze for lung (**a**) and BAL (**b**) PDL1 levels. Mice from Figure 1 were analyzed for BAL Angpt2 (**c**) and PDL1 (**d**) levels. Male C57Bl/6 mice received intranasal administration of IAV H1N1 PR8 (800 pfu/mouse) on Day0 (**e**–**h**). On Day6 and 8, IAV mice were SQ injected with 8 mg/kg of anti-mouse PDL1 antibody (brown columns), or an equivalent volume of PBS (red columns). Lung tissue and BAL fluids were collected on Day9 to analyze for lung PDL1 levels (**e**), BAL Angpt2 levels (**f**), BAL total protein (**g**) and WBC (**h**). *n* = 3–8 per group, presented are mean +/− SEM. Lines indicate levels of analytes detected in control (saline to lung) mice. * for *p* < 0.05 by *t*-test with Welch correction (unequal variances) between groups indicated (**a**,**b**,**e**–**h**) or with control group, plotted as Day0 (**c**,**d**). NS - differences between the groups are not significant.

To determine whether Angpt2 release in IAV-infected mice is regulated via the PDL1-dependent pathway, we first checked for the correlation between Angpt2 and PDL1 levels in IAV-infected mice. Similar to Angpt2 BAL levels (Figure 7a), PDL1 levels in lung and BAL were dramatically increased in response to the infection and decreased in response to the ASC secretome administration (Figure 8a,b). We next checked for the temporal correlation of changes in PDL1 and Angpt2 levels. Both PDL1 and Angpt2 BAL levels demonstrated detectable increases during the early phase of IAV infection, followed by the dramatic increases in the lung injury phase (Figure 8c,d).

To ascertain whether the suppression of PDL1 level by ASC-CS is causative to the suppression of Angpt2 release and lung injury in general, we neutralized PDL1 using the same time frame as was employed to study the effect of ASC-CS administration. Mice receiving anti-mouse PDL1 antibody at Day6 and 8 post-infection demonstrated dramatic decrease in lung PDL1 levels, proving that subcutaneous dose of 8 mg/kg was sufficient to pharmacologically ablate PDL1 in lung (Figure 8e). However, IAV-induced Angpt2 level was only non-significantly decreased in response to the late-phase PDL1 ablation. Most importantly, analysis of ARDS indices revealed only modest trend to mitigation of lung injury by the late administration of PDL1-blocking antibody.

## 4. Discussion

We had shown previously that murine ASC-CS is able to limit lung injury in mice challenged with LPS [31]. Here, we report the development of a concentrated preparation of human ASC-CS for the purpose of treatment of lung injury induced by viral infection. SQ administration of CS approximate dose equivalent to the secretome of 1.6 million human ASC significantly suppresses IAV PR8-induced lung injury, when first administered six days post-infection. This is dramatically different from the earlier reported absence of prophylactic and therapeutic efficacy of human BM-MSC in the same model [19,20]. The apparent difference is that use of CS allows for administration of a secretome product derived from a higher equivalent cell dose compared to the dose used in BM-MSC studies (0.25–0.5 million [19,20]) and with potential for immediate bioavailability of secretome.

Our data clearly show that application of the immunomodulatory ASC-CS material early in the course of lung infection (viral stage) appears to be detrimental to mice. Accumulating data of literature recognize the necessity to limit the use of immunomodulators to the later stages of viral ARDS, rather than indiscriminately include the initial stage of infection when immune suppression may aggravate the disease [32]. This approach is currently tested in the clinical trial of early vs. late dexamethasone in COVID19 (NCT 04530409). Our preclinical data suggest that critical regard to the timing of immunomodulatory intervention is likely to benefit treatment of the other types of viral ARDS, including influenza-induced lung injury, for which application of corticosteroids was earlier found to be of no benefit or even detrimental [33,34]. In our study, late administration timed at the start of respiratory decline was able to limit ARDS severity, in contrast to the early administration timed at viral replication stage. Late administration also warranted relevance of the proposed approach to the clinical scenario of treatment of patients presenting with the developing viral pneumonia.

The efficacy of therapy was specifically tested in both male and female, as accumulating data of literature show that clinical response to antivirals, rates of IAV hospitalization and ARDS development, and efficacy of ARDS management differ significantly in men and women. In IAV-positive patients, slower alleviation of symptoms and lower reduction in viral loads are reported in females [35]. Although reports are limited, available data suggest that likelihood of severe seasonal influenza is higher in post-adolescent females than in males [36]. Several factors other than physiological differences can account for female vulnerability to severe influenza. Professions influencing likelihood of exposure to IAV, such as health and childcare providers and child educators, lower vaccine acceptance and more frequent inappropriate prescription of antivirals in females are likely to affect incidence, duration, severity and fatality rates of influenza (reviewed in [25]). Accumulating data of evidence show that females are both predisposed to lung injury and are subjected to medical procedures leaving their lung unprotected during lung injury management. A report on trauma patients requiring ICU admission had shown that female lung is more prone to ARDS development independently of the older age and injury patterns [37]. More strikingly, recent study had shown that females with severe confirmed ARDS have a higher ICU mortality risk, and that females with ARDS receive higher tidal volumes and higher plateau and driving ventilating pressures compared with males [38]. Altogether, these data suggest that there is a particular need to develop therapy effectively targeting severe influenza and ARDS in females. In our model, infection of males and females with similar dose of virus resulted in comparable increases in WBC BAL counts, BAL protein content and water content in lung; however, MPO levels were more than 50 times higher in female BAL when compared to male. Interestingly, data of literature report both increased [39] and reduced [40] H1N1 PR8 influenza severity in female mice.

Sex differences can also affect the properties of the cell-derived therapeutics. Studies in rat [41] and porcine [42,43] MSC show that female cells produce less pro-inflammatory factors and more growth factors than male cells. Analysis of genes expressed by ASC from healthy non-obese human donors confirms sex effect on the cell immunomodulatory capacity [44]. However, when human MSC are discussed, one has to remember that donor’s age [45], health status, body mass index [46] and cell culturing conditions [47,48] are likely to affect MSC paracrine patterns more than an individual donor’s sex would. The objective of the current study was not to analyze possible differences in the therapeutic potency of male and female ASC, but rather to pre-clinically validate secretome of a particular donor generated with a particular cGMP-compliant protocol in the context of viral lung injury. Of note, choice of the particular ASC donor for production purposes was based on testing several donors’ ASC for cell proliferation, surface marker profile, differentiation potential, and levels of secreted factors. Generation of cGMP Master Cell Bank from the chosen donor, and its regulatory-appropriate testing as part cGMP-compliant protocol development are required for transitioning the candidate therapeutic to clinical trials. Assessment of the chosen test article in the context of IAV-induced lung injury revealed significant suppression of inflammation and hyperpermeability in the mixed gender group, with therapeutic effect of ASC-CS more pronounced in the female mice. Tests of ASC-CS preparations from other donors’ ASC were beyond the scope of the present study.

ASC-CS has several potential advantages versus cell therapy, including potential ease of storage and handling. In the present study, we show that ASC-CS preparation stored for three years at −80 °C, the longest stored comparative ASC-CS at the time of the present study, exhibited ARDS-mitigating properties non-inferior to the properties of the freshly prepared and briefly frozen ASC-CS, which represents a significant advantage versus cells that must be stored at <(−135) °C. Moreover, ASC-CS preparation was effective when administered immediately after thawing, whereas cells typically require complicated thawing and washing procedures prior to administration. Testing additional storage conditions and time points will be part of stability studies conducted prior to and in parallel with clinical trial of ASC-CS.

To explore the potential to improve therapeutic efficacy of ASC-CS, we primed ASC with inflammatory mediators TNFα and IFNγ. For a decade, priming approaches were successfully used to improve MSC anti-inflammatory and trophic activities in vitro and in inflammatory models such as model of induced colitis (reviewed in [49]). Our method of secretome generation allows for easy removal of inflammatory mediators used for priming, providing a path for a cGMP-compliant product of induced potency. Assessment of the primed ASC-CS effect in the model of male IAV infection resulted in significant attenuations of protein leak and WBC infiltration, unattained earlier in male mice treated with the original ASC-CS.

To gain understanding of ASC-CS effect on pulmonary barrier, and, in particular, microvascular permeability, we assessed the levels of an endothelium-secreted barrier disruptor Angpt2. The levels of Angpt2 were long shown to be associated with vascular injury and leak [50,51,52] and ARDS severity [53]. Dramatic release of Angpt2 to BAL was observed during lung injury stage of infection, with marked attenuation of released levels by the original and primed ASC-CS. Angpt2 is known to act as an antagonist of the endothelial regulatory receptor Tie-2 during inflammation [54]. Tie2 is a tonically phosphorylated receptor tyrosine kinase, which in non-lymphatic endothelial cells is finely regulated by its agonist Angpt1, antagonist Angpt2, and vascular endothelial protein tyrosine phosphatase VE-PTP [55]. Reduced Tie2 mRNA expression was linked to states associated with increased vascular leak, including murine lung influenza infection [56]. We have not observed decreased Tie2 protein expression in murine lung during lung injury stage; this disparity may be due to the differences in Tie2 mRNA and protein stability during lung injury. In infected lung, we observed significant decrease in Tie2 phosphorylation, concomitant with the loss of pulmonary barrier function. Consistent with the proposed barrier-enhancing role of p-Tie2, Tie2 agonist vasculotide was recently shown to improve survival and decrease vascular leak in influenza-infected mice in a manner independent of neutrophil recruitment or viral clearance regulation [57]. Our data show that, despite marked Angpt2 downregulation, ASC-CS beneficial effect is not exerted via increase in Tie2 phosphorylation, possibly because attenuated Angpt2 levels remain above the threshold level sufficient to induce Tie2 dephosphorylation. Involvement of other Angpt2 downstream targets in therapeutic response to ASC-CS stays beyond the scope of this study.

To gain understanding of processes driving modulation of Angpt2 during therapeutic response to ASC-CS, we assessed potential involvement of a recently identified PDL1/Angpt2 axis. Recent work has shown that PD1/PDL1 pathway, known as immune checkpoint pathway, has function in endothelium where it regulates endothelial Angpt2 release [29]. In the murine model of shock/sepsis-induced ARDS, mice deficient for PD1 or PDL1 manifested decreased BAL protein extravasation in response to injurious stimuli; suppressed barrier dysfunction was linked to the decreased Angpt2 production by endothelium. Endothelial role of PDL1 was further corroborated when intravenous, but not intratracheal delivery of PDL1 siRNA effectively reduced lung edema and inflammation in the same model of indirect ARDS [58]. Assessment of correlation of PDL1 and Angpt2 levels in infected and therapy-treated mice supported the perspective link between modulation of PDL1 levels and Angpt2 release. To prove causative relationship between ASC-CS induced downregulation of PDL1 and decrease in Angpt2, we neutralized PDL1 using the same therapeutic intervention scheme as was used for ASC-CS administration. Injection of anti-mouse PDL1 antibody at D6 and D8 post-infection resulted in more dramatic attenuation of lung PDL1 levels than those achieved by ASC-CS. Surprisingly, the suppression of Angpt2 release did not achieve significant level; more importantly, the effect on both IAV-induced barrier permeability and inflammation was only marginal. Altogether, these data suggest that suppression of PDL1 by ASC-CS does not play a critical role in the therapeutic effects of ASC-CS.

Summarizing, this study served as a preclinical validation of ASC-CS from a selected donor generated with a cGMP-compatible protocol developed by Theratome Bio, Inc. The protocol allows for generation of multiple doses using ASC from single donor, sufficient for clinical treatment of multiple patients. Similar approach is commonly used in clinical manufacture of cellular products, where single donor cells following culture expansion generate multiple doses of the donor cell bank (Remestemcel-L Manufacturing Process [59]). The manufacture of multiple doses of ASC-CS from ASC of a single donor facilitates development of a consistent product and minimizes comparability testing required when multiple cell donors are utilized. Challenges of clinical manufacture of secretome product include development of the analytical methods to ensure minimal lot-to-lot variations in identity, purity, and the application-relevant potency [60]. Ideally, in vitro potency assessment methods should be first validated for their ability to reflect differences in vivo potencies of the tested secretome preparations. We have recently reported that an in vitro assay of endothelial monolayer permeability is sensitive to the species-specific origin of secretome [61] and therefore is not suited to reflect potency of human ASC-CS preparations tested in the murine models of lung injury. Mechanistic insights obtained in this study suggest that regulation of VEGF/Angpt2 axis by ASC-CS may be used as a basis for the development of a desired in vitro assay translatable to both murine models and clinical application of ASC-CS.

## 5. Conclusions

We have developed a clinically translatable preparation of concentrated ASC secretome with the ability to mitigate lung injury when applied during lung injury development stage. This therapeutic product principally differs from the standard-of-care antivirals with their narrow therapeutic window and susceptibility to acquisition of drug resistance. Immune priming of secretome-generating ASC enhanced secretome ability to limit lung injury, providing basis to improving product potency. Beneficial effects of secretome were observed concomitantly with the decreased release of barrier disruptor Angpt2; however, levels of p-Tie2 were not augmented in response to ASC-CS therapy, indicating that other downstream targets of Angpt2 mediate ASC-CS effect on barrier function. Late-phase neutralization of PDL1 resulted in non-significant attenuation of Angpt2 levels and marginal effects on hyperpermeability and inflammation, suggesting that ASC-CS-mediated downregulation of PDL1 does not contribute significantly to the beneficial effects of ASC-CS.

## 6. Patents

The patent for ASC and ASC conditioned media application to treat ARDS, SARS and MERS is currently owned by Indiana University (NVB is one of the inventors).

## Data Availability

All data are contained within the presented article.

## Abbreviations

Angpt2	Angiopoietin 2
ARDS	acute respiratory distress syndrome
ASC	adipose stromal cells
BAL	bronchoalveolar lavage
BM	bone marrow
cGMP	current Good Manufacturing Practices
CS	concentrated secretome
HPAEC	human pulmonary artery endothelial cells
IAV	influenza A virus
ICU	intensive care unit
MPO	myeloperoxidase
MSC	mesenchymal stromal cells
PDL1	programmed cell death ligand
SQ	subcutaneous
WBC	white blood cells
